# Radiotherapy plus CAR-T cell therapy to date: A note for cautions optimism?

**DOI:** 10.3389/fimmu.2022.1033512

**Published:** 2022-11-17

**Authors:** Tian Huan, Hongbo Li, Bin Tang

**Affiliations:** ^1^ Department of Rehabilitation Medicine, Jinhu County People’s Hospital, Huaian, Jiangsu, China; ^2^ School of Life Sciences, Jiangsu University, Zhenjiang, Jiangsu, China

**Keywords:** radiotherapy, immunotherapy, CAR-T cell therapy, tumor microenvironment, radioresistance

## Abstract

Radiotherapy (RT) is a traditional therapeutic regime that focuses on ionizing radiation, however, RT maintains largely palliative due to radioresistance. Factors such as hypoxia, the radiosensitivity of immune cells, and cancer stem cells (CSCs) all come into play in influencing the significant impact of radioresistance in the irradiated tumor microenvironment (TME). Due to the substantial advances in the treatment of malignant tumors, a promising approach is the genetically modified T cells with chimeric antigen receptors (CARs) to eliminate solid tumors. Moreover, CAR-T cells targeting CSC-related markers would eliminate radioresistant solid tumors. But solid tumors that support an immune deserted TME, are described as immunosuppressive and typically fail to respond to CAR-T cell therapy. And RT could overcome these immunosuppressive features; thus, growing evidence supports the combination of RT with CAR-T cell therapy. In this review, we provide a deep insight into the radioresistance mechanisms, advances, and barriers of CAR-T cells in response to solid tumors within TME. Therefore, we focus on how the combination strategy can be used to eliminate these barriers. Finally, we show the challenges of this therapeutic partnership.

## 1 Introduction

Radiotherapy is the localized cancer treatment of cancer patients through high-energy radiation. The principle is to induce double-stranded DNA damage, single-strand breaks, incomplete repairs, and chromosomal aberrations in cancer cells to accomplish local tumor control and reduce the outcome of disease transmission (1). In addition to the direct destruction of cancer cells, the effects of RT on the tumor microenvironment are manifested in terms of responsiveness and immune sensitivity. On the one hand, RT-induced tumor cell death leads to systemic antitumor effects by releasing pro-inflammatory cytokines, chemokines, and tumor antigens, which in turn trigger the potential for adaptive and innate immune responses (2, 3).

**Figure 1:**
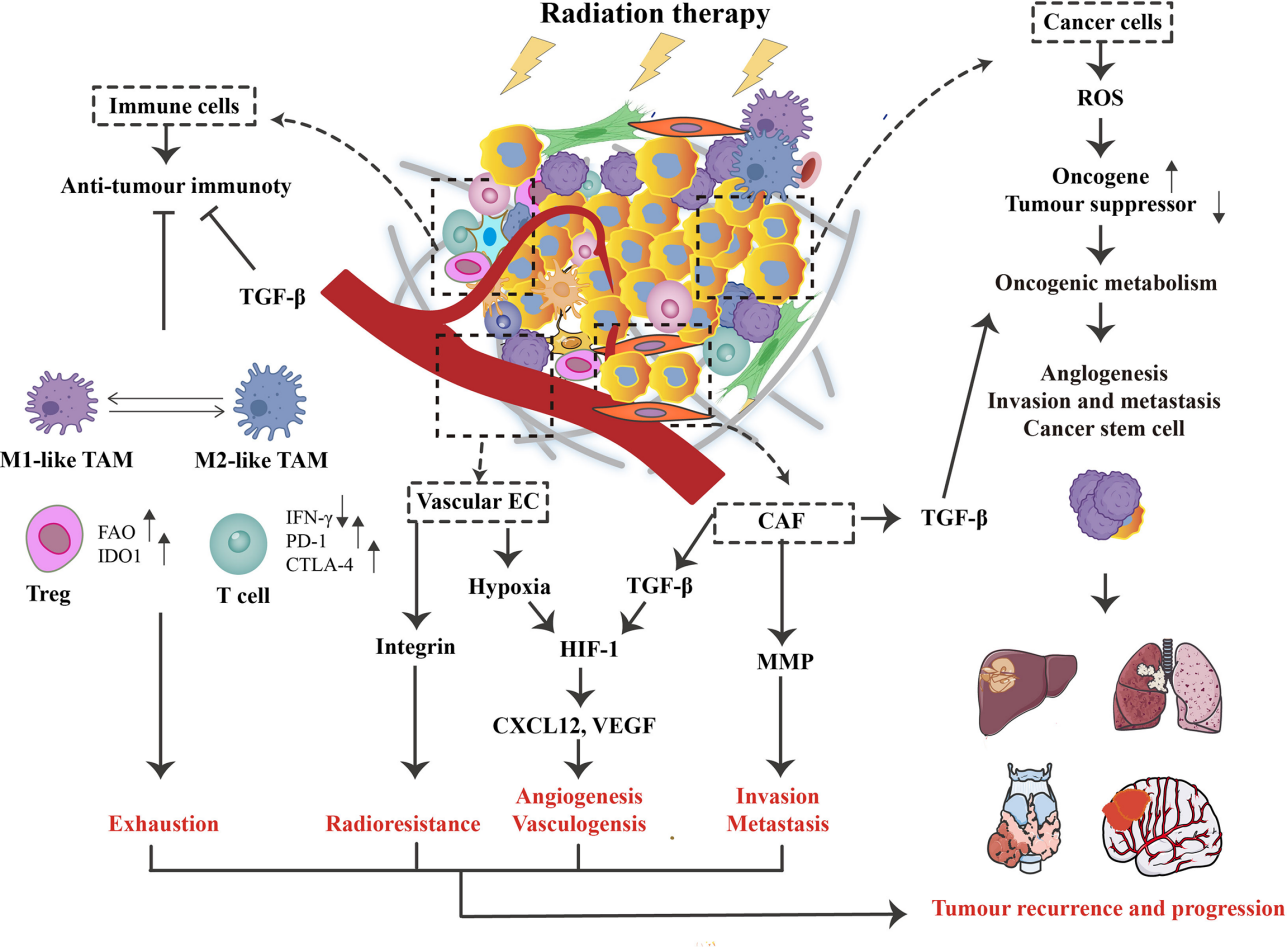
The role of radiotherapy(RT) on the tumor microenvironment (TME). Concerning cancer cells, RT can promote the generation of reactive oxygen species (ROS), causing activated oncogenes and inactivated tumor suppressors. This process supports oncogenic metabolism, increases tumor aggressiveness, and ultimately leads to recurrence and distant metastasis. In addition, RT triggers various changes in TME, such as hypoxia and immune responses. For intense, TGF-β can influence cancer cells and CAFs, enhancing tumor immune escape and associated activation of HIF-1 signaling. At the same time, vascular endothelial cells (ECs) are damaged, contributing to hypoxia and promoting the HIF-1 signaling, further stimulating the expression of vascular endothelial growth factor (VEGF) and CXCL12. Although RT activates anti-tumor immune responses, this signaling is often inhibited by tumor escape mechanisms such as TAM, Treg, and the PD-1/PD-L1 signaling pathway, which is relatively low radiosensitive compared to other lymphocyte subpopulations. Ultimately, these RT-induced changes in TME may contribute to the poor therapeutic effect of RT on patients by facilitating exhaustion, angiogenesis, invasion, and radioresistance.

On the other hand, radioresistance, a side effect of RT, is mediated by multiple mechanisms in the tumor microenvironment (TME) (4). TME has many limitations and biochemical characteristics, such as acidic extracellular pH, hypoxia, excessive glutathione, etc. It consists of four components: (1) an immune component consisting of several different immune cells, such as T cells, natural killer (NK) cells, tumor-associated macrophages (TAMs), and dendritic cells (DCs); (2) vascular component consisting of blood and lymphatic endothelial cells. (3) the extracellular matrix (ECM) fraction formed by complex collagen fibers and other glycoproteins; and (4) the stromal fraction composed of cancer-associated fibroblasts (CAFs) and mesenchymal stem cells (MSCs) (5, 6). The sensitivity of various immune cells to irreversible damage induced by RT, such as cell death and chromosomal instability, is different. For example, regulatory T cells (Tregs) are more resistant to radiation than any other T cell population, while NK cells and B lymphocytes are the most radiation-sensitive immune cells (7). Secretion of type I interferons (IFN-1) may cause the upregulation of programmed death-ligand 1 (PD-L1) in tumor and immune cells. Upregulation of PD-L1 expression on tumor cells hinders the anti-tumor function of activated T cells and NK cells. T cells can also overexpress PDL-1 after radiation and help prevent tumor cell recognition. Also, activated transforming growth factor-β (TGF-β) suppresses the radiosensitivity of tumor cells and enhances immunosuppression by reducing CD8^+^ T cell toxicity, promoting Treg transformation, and inhibiting NK cell proliferation. Concurrent radiation damage can enhance pro-inflammatory responses after irradiation and recruit CAFs. Activated CAFs may secrete TGF-β and matrix metalloproteinases (MMPs), extracellular matrix modulators, to promote conversion to radioresistant cancer stem cells (CSCs) (8, 9). Radioresistance is the most crucial cause of radiotherapy failure (**Figure 1**). Thus, ultimate tumor control may depend on the balance of immunostimulatory and immunosuppressive signals generated within the tumor.

In recent years, Chimeric antigen receptor (CAR)-T cell therapy has been considered one of the most successful approaches in tumor immunotherapy, particularly in treating hematological tumors. The principle is to engineer synthetic receptor CARs to redirect T cells to recognize and eradicate tumor cells expressing specific target antigens independent of the MHC receptor, which causes the activation of powerful T cells and a more robust anti-tumor response *in vivo* (10). For certain types of solid tumors, such as melanoma, CAR-T can significantly improve survival conditions without interruption and induce long-term durable remissions, meaning that some patients with solid tumors can also derive lasting and stable benefits from CAR-T cell therapy (11). However, the number of patients with durable responses to solid tumors alone in the clinic is only a minority. CAR-T cells still face many dilemmas in treating solid tumors. Therefore, there is an urgent need to expand the beneficial range of immunotherapy and identify appropriate patient choices. Low-dose RT can sensitize antigen-negative tumor cells to CAR-T cells induced elimination (12). Combining these two therapeutic modalities, therefore, holds clinical therapeutic promise. Indeed, combination therapy has produced exciting results for radioresistant tumor cells in clinical and preclinical trials.

This paper reviews the mechanisms of tumor radioresistance in irradiated TME. Here, we also summarize the role of CAR-T cells in the solid tumor microenvironment and experimental data on combination therapy, which provide a basis for further exploration of combination therapies to improve the outcomes of tumor radioresistance.

## 2 Mechanisms of radioresistance in irradiated TME

In irradiated TME, there are dynamic interactions and crosstalk between these components within TME. Changes in these TME mediate various immunosuppressive mechanisms or the development of immune suppressor cells to promote tumor radioresistance.

### 2.1 Hypoxia-mediated radioresistance

**Figure 2:**
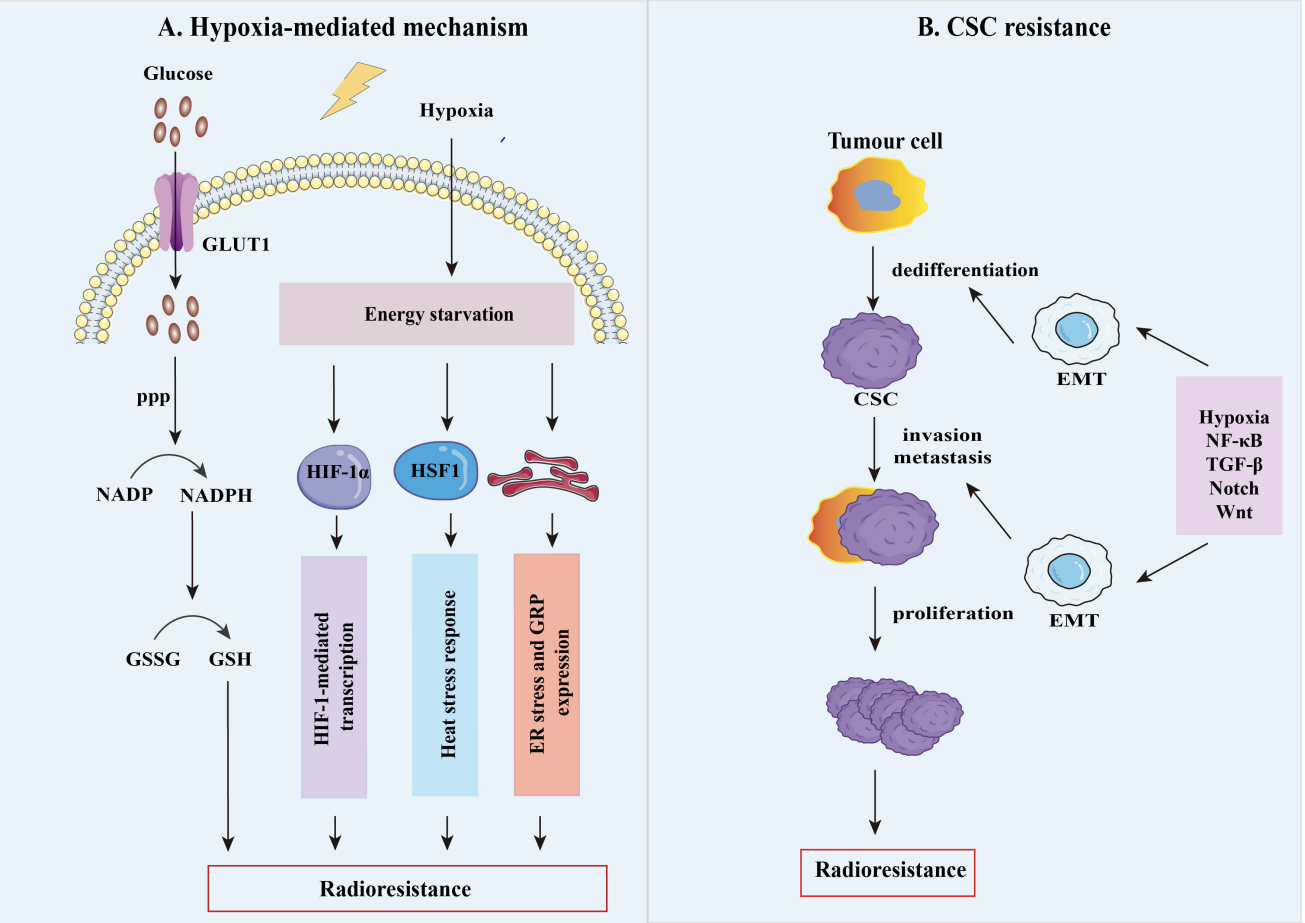
The mechanisms of tumor radioresistance. **(A)** Tumor hypoxia is thought to be an important factor responsible for the radiation resistance of solid tumors, as oxygenation is vital for the efficacy of RT. Hypoxia has a fundamental impact on the resistant phenotype of tumors by upregulating various factors such as HIFs, glucose-regulated protein (GRP), heat shock transcription factor 1(HSF1), heat shock protein (HSP), which result in the radioresistant hypoxic tumors. **(B)** Cancer stem cells (CSC) are highly plastic cell groups capable of acquiring different phenotypes and cellular states, evading treatment, and enhancing their ability to grow tumors potently. After irradiation, tumor stem cell plasticity is mainly controlled by TME hypoxic conditions and signaling, including nuclear factor-κB (NF-κB), transforming growth factor-β (TGF-β), Notch, and Wnt. Meanwhile, epithelial-mesenchymal transition (EMT) plays an essential metabolic reprogramming role in this process, allowing cancer cells to be dedifferentiated into CSCs to reach distant metastatic sites.

The majority of solid tumors exhibit hyperoxygenation, which results in areas of permanent or transient hypoxia being developed. Cellular adaptation to these hypoxic conditions is mediated through a family of hypoxia-inducible transcription factors (HIFs) (13, 14), which can regulate various genes to promote or maintain glycolytic metabolism (**Figure 2A**). Aerobic glycolysis is the most critical metabolic feature in the Warburg phenotype to promote survival and long-term maintenance for tumor cells (15). This process, composed of genes involved in glucose transporter 1 (GLUT1) and HIF-1, results in increased intracellular glucose and glucose-6-phosphate levels. As glucose-6-phosphate is the substrate of the pentose phosphate pathway, it is in charge of the biological production of the antioxidant NADPH, glutathione, high-speed ATP production, and the accumulation of lactic acid (16, 17).

Heat shock protein (HSP) serves as a molecular chaperone rapidly upregulated when exposed to harmful stimuli under oxidative stress, contributing to the correct folding of proteins, degradation, and removal of denatured proteins. It is well known that the heat shock transcription factor 1 (HSF1) can confer tumor cell radioresistance by upregulating HSP27, HSP70and HSP90 protein levels, suppressing post-radiation cell apoptosis, and correlates with poor prognosis in patients (18). HSP27 can inhibit apoptosis by blocking the activation of cytochrome c-induced caspases during different stages. Indirectly inhibiting cytochrome c release at the pre-mitochondrial level through its action on a BH3-only member of the Bcl-2 family proteins (Bid), reactive oxygen species (ROS), or filamentous (F-actin) and at the post-mitochondrial level through the cytosolic cytochrome c sequestration (19, 20). HSP70, the decisive negative regulator, can prevent mitochondrial membrane permeability by blocking Bax transport at the mitochondrial level. In contrast, at the post-mitochondrial level, HSP70 accomplishes its task of blocking apoptosis by interaction with Apaf-1 and AIF or by the protection from caspase-3 cleavage of essential nuclear proteins (21).

In addition, cell cycle status also determines tumor radiosensitivity under hypoxic conditions. Other studies demonstrated that tumor cells are more resistant to radiotherapy in the cellular cycle’s late S and G0 phases, while cells in the G2/M phase are more sensitive (22, 23). Zhu Y et al. illustrated that if the same level of cell-killing effect is necessary, the radiation dose of late S-phase cells is about 1.3-2.0 times that of G1-phase cells (24). Knock-down of HSF1 by small interfering RNAs transfecting colorectal cancer cells HCT116, the comet assay results showed that lack of a functional HSF1 was unable to arrest in the G2-phase of the cycle and reduced the capacity of double-stranded DNA break repair after exposure to ionizing radiation (25). Indeed, hypoxia-induced cell cycle arrest is accompanied by a decreased activity of certain cyclin-dependent kinase (CDK) complexes leading to inhibition of cell cycle progression. CDK activity is regulated by CDK-cyclin inhibitors such as p27^Kip1^ and p21^waf1^, and dysregulation of CDK activity is a common characteristic of numerous cancers (26). p27^Kip1^ is reported to function in the cell cycle at the G1checkpoint when knockdown of HIF-1α expression resulted in a significant reduction in the level of p27^Kip1^ as well as showed a decrease and increase in the proportion of G1- and S-phase cells, indicating the dependency of p27^Kip1^expression on HIF-1 (24). Similarly, there is positive interactive feedback of p21^waf1^ and HIF-1α, which induces glycolysis through upregulating Glut1 and LDHA expression and increases the radioresistance of GBM (27). Furthermore, hypoxia-induced radioresistant prostate cancer cells (LNCaP and C4-2B cell lines) were due to HIF-1α-mediated expression of β-catenin nuclear translocation, which resulted in cell cycle alterations, reduced apoptosis, and improved nonhomologous end joining in DNA break repair after radiation (28).

Altogether, all this implies that hypoxia plays a critical role in high radioresistance, which is granted by multiple mechanisms like HIF-1-induced gene transcription, reprogramming of energy metabolism, heat stress response, and cell cycle changes.

### 2.2 Generation of radioresistant CSC-like cells

A primary element of radioresistance is the role of the CSCs population in a tumor, which explains why tumor cells metastasize and relapse (29, 30) (**Figure 2B**). CSCs are a proportion of cancer cells within solid tumors with self-renewal and tumor maintenance properties. Increasing evidence proved that CSCs contribute to radioresistance through multiple molecular mechanisms, including activation of survival signaling pathways (TGF-β, Wnt, and PI3K/Akt/mTOR, etc.) and the epithelial-mesenchymal transition(EMT) process, which have a crosstalk circuit (31). In breast tumors, Liu et al. documented that TGF-β1 expression was positively related to macrophage abundance and responsible for EMT and CSC by analyzing the TCGA database (32). Accumulating evidence has demonstrated that RT-mediated EMT Can result in the generation of CSCs generally radioresistance. Most tumor cells are eradicated by the induction of apoptosis or mitotic death after RT. However, a small population of non-stem cancer cells (namely non-CSCs) can show the radioresistant features and dedifferentiate and transform them into CSCs through RT-mediated EMT. Newly generated CSCs from non-CSCs, in conjunction with intrinsic CSCs, ultimately contribute to tumor recurrence and metastasis (33, 34). For example, exposure of non-CSCs to ionizing radiation, isolated from hepatocellular carcinoma cell lines (HepG2 and Huh7), demonstrated stem cell-like properties like more sphere formation and stemness gene expression for radioresistance (35). Furthermore, several reports to date have demonstrated that CD133, CD44, CD44^+^/CD24^-^ and CD34^+^/CD38^-^ can serve as specific surface markers for CSCs in different human tumors (36, 37).

These results strongly indicate that CSCs are a promising therapeutic target for resistance to RT.

### 2.3 Radioresistance of immune cells

**Figure 3:**
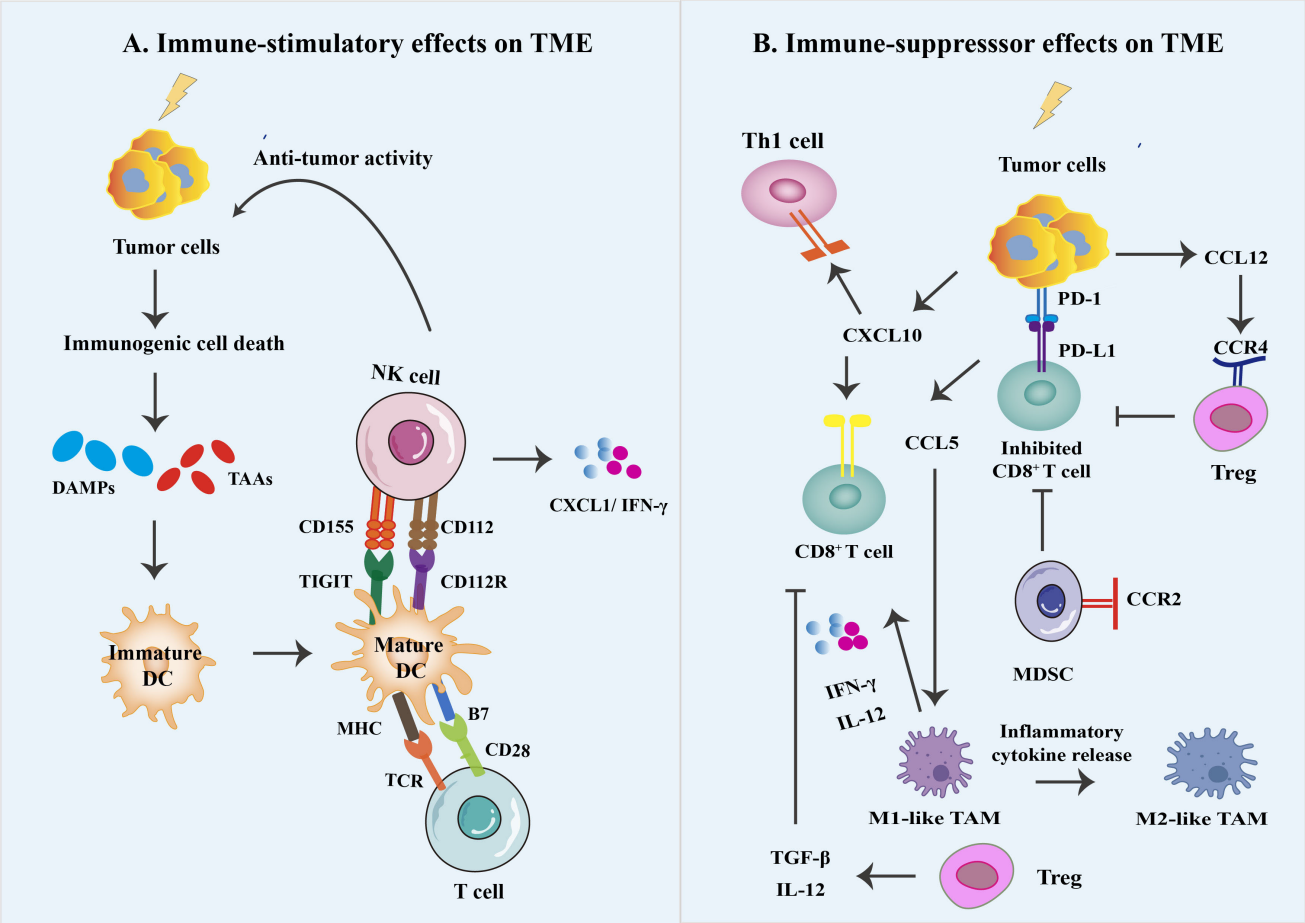
Different immune effects on the irradiated tumor microenvironment. **(A)** The generated cellular stress and death mediate immunogenic cell death (ICD) by the generation of damage-associated molecular patterns (DAMPs) and their corresponding pattern recognition receptors (PRRs). ICD can trigger a series of antitumor immune responses. This process requires DAMPs signaling through various PRR types, activating DC, NK cells, and CD8^+^ T cells and increased production of associated inflammatory cytokines. Eventually, inflammatory signaling is increased, starting DCs and maturing them into potent antigen-presenting cells APC. **(B)** RT can produce a variety of chemokines that promote and antagonize anti-tumor responses, e.g., T cells and M1 macrophages can be recruited *via* CXL10 and CCL5, respectively, to eliminate tumors. In contrast, myeloid-derived suppressor cells (MDSC) can have immunosuppressive effects through radiation-induced recruitment of CCL12 expression. In addition, small doses of radiation delivered during conventional fractionated radiation therapy are thought to contribute to the accumulation and immunosuppressive effects of immunosuppressive cell types in the TME, such as myeloid-derived suppressor cells (MDSCs) and M2 macrophages to suppress antitumor immunity and PD-1/PD-L1 suppressor signaling to suppress antitumor immunity.

As a double-edged weapon, RT may activate or inhibit the TME immune response in various conditions. Inflammatory signaling occurs after ionizing radiation by activating cell survival pathways and stimulating the innate immune system (**Figure 3A**). These include IL-1 and TNF inflammatory cytokine signaling and recruitment of immune cells *via* endothelial cells expressing intercellular adhesion molecule 1 (ICAM1), vascular cell adhesion molecule 1 (VCAM1), and E-selectin (4). Meanwhile, the generated cellular stress and death mediates immunogenic cell death (ICD) by the generation of damage-associated molecular patterns (DAMPs) and their corresponding pattern recognition receptors (PRRs) (38). ICD is thought to be crucial in triggering a potent antitumor immune response, a process that requires DAMPs signaling through various PRR types, such as HSP90 or calretinin surface exposure leading to CD91-mediated phagocytosis, which is expressed on diverse innate immune cells; TLR2 and TLR4 activation by HGMB1 release, resulting in DC activation and increased production of associated inflammatory cytokines; secretion of ATP activates purinergic receptors P2Y2 and P2X7, which have extensive immunostimulatory effects among DCs, NK cells, T cells, and macrophages (39, 40). Eventually, inflammatory signaling is increased, activating DCs and maturing them into potent antigen-presenting cells APC.

Even though RT enables the immune system to act against cancer cells through ICD, it may still be limited through changes in the percentage of immune cells in the TME due to a relative increase in radioresistant suppressor cell types such as Treg, MDSCs, and TAM within the TME (41). It has been reported that RT-induced inflammatory signaling usually triggers counter-regulatory immunosuppressive mechanisms (**Figure 3B**). The modulation is mainly through the alteration of multiple cytokines signaling, including tumor necrosis factor (TNF), interleukin-1β (IL-1β), interleukin-10 (IL-10), and transforming growth factor beta (TGFβ) (4, 42). For example, T cell activation requires costimulatory signals achieved through interacting CD28 expression on T cells with CD80 and CD86 expression on APC. However, Treg can express high levels of cytotoxic T lymphocyte antigen 4 (CTLA-4), with a greater affinity for CD80 and CD86 compared to CD28, which competitively provide ineffective costimulatory signals of T cells, resulting in Treg-induced immune suppression (43).

In comparison to other T cell populations, Tregs (CD4^+^CD25^+^ T cells) and immunosuppressive MDSCs are more radioresistant. Shi et al. evaluated the impact of 10, 20, or 30 Gy local irradiation in cervical cancer patients. They found that the number of CD8^+^ T cells has dramatically reduced, whereas not affecting Tregs. The accumulation of T cells after ablative radiotherapy and exhaustion of CD8^+^ T cell infiltration is an essential mechanism of radioresistance (44). Likewise, MDSCs have demonstrated the accumulation in the TME and inhibit CD4^+^ and CD8^+^ T cell activation. Studies have shown that MDSC and TAM can express high levels of arginase-1 (Arg-1), which reduces the pool of arginine for T-cell activation, and they also sequester a cysteine that is essential for T-cell proliferation, thereby restricting cysteine availability and consequently disrupting the T-cell receptors (TCRs) by generating ROS (45). Additionally, studies have reported PD-1 expression increases on T cells and PD-L1 on tumor cells after RT, causing inactivation and depletion of CD8^+^ T cells, inhibiting the antitumor immune response and developing radiotherapy tolerance (46, 47).

## 3 CAR-T cell therapy

Chimeric antigen receptor T cells (CAR-T cells) have achieved promising outcomes in patients with hematologic malignancies However, it still has some challenges that need to be resolved for solid tumors. We detail a series of considerations for the improvement of the CAR-T cell approach in order to make CAR-T cell therapy more widely available (**Table 1**).

**Table 1:** Safety strategies for overcoming the suppressive tumor microenvironment by CAR-T cells.

Tumor-associated antigens	Overcoming strategy	Reference
CD19	CAR-T cells expressing an oxygen sensitive subdomain of HIF1α	Antonana-Vildosola et al. (5)
CAIX	CAR-T cells Targeting hypoxia downstream signaling protein, CAIX	Cui et al. (48)
CD19, HER2, AXL	CAR-T cells expressing an oxygen-dependent degradation domain (ODD)	Liao et al. (49)
Nectin4/FAP	CAR-T cells targeting cancer-associated fibroblasts	Zhou et al. (50)
αvβ3 integrin ICAM-1 VCAM-1	CAR-T cells targeting the tumor vasculature	Uddin et al. (51) Vedvyas et al. (52) Ma et al. (53)
B7-H4 VISTA TIM4	CAR-T cells targeting tumor associated macrophages and myeloid suppressor cells	MacGregor et al. (54) Date et al. (55)

CD19, B-lymphocyte surface antigen; CAIX, carbonic anhydrase IX; HER2, human epidermal growth factor receptor 2; AXL, AXL receptor tyrosine kinase; Nectin4, human tumor cell marker PVRL4; FAP, fibroblast activation protein; αvβ3 integrin, transmembrane cell adhesion receptor; ICAM-1, intercellular adhesion molecule-1; VCAM-1, vascular cellular adhesion molecule-1; B7-H4, B7 family ligands 4; VISTA, V-domain Ig-containing suppressor of T cell activation; TIM4, T-cell immunoglobulin mucin protein 4.

### 3.1 Design of chimeric antigen receptors

CARs are modular synthetic receptors that can confer target antigen specificity. For structure, it consists of four main components: (i) the antigen-binding domains are derived from a single-chain variable fragment (scFv), (ii) the hinge or spacer region, (iii) the CD3ζ, CD8α, or CD28 transmembrane domain, and (iv) one or more intracellular signaling domains (10). First-generation CARs could initiate a cytotoxic antitumor response in grafted T cells when T cells are activated. They can eradicate tumor cells effectively through secreting granzyme and expressing FasL, a tumor necrosis factor-related apoptosis-inducing ligand (TRAIL). However, the CAR construct has become increasingly more specific and sophisticated since our knowledge of molecular biology and synthetic biology about T cell activation and TME has improved. The second generation of CARs only has two costimulatory molecules (CD28, CD137, CD278, etc.) to activate the entire physiological T cell and enhance proliferation. The third generation of CARs includes CD3ζ and two costimulatory domains. Fourth-generation and next-generation CAR-T cells, which can improve cytotoxicity and modulate the immune system, consist of a nuclear factor of activated T cells (NFAT) domain, a suicide gene, or signaling domains from proinflammatory cytokines, like Interleukin-7 (IL-7) or IL-18, etc (56).

### 3.2 Limitations of CAR-T cell therapy

Although the principle of CAR-T cells is to achieve tumor lysis through direct interaction between T cells and tumor cell surface-specific antigens, tumor heterogeneity is challenging for CAR-T cells (57). In recent years, researchers have attempted to develop a new generation of CAR-T cells in multiple ways to overcome a wide range of TME inhibitors. The current preclinical studies (**Table 1**) will be expected to provide information about the efficacy and safety of CAR-T cell therapy against solid tumors to overcome the barriers of TME.

#### 3.2.1 CAR-T cell trafficking and infiltration

The effectiveness of CAR-T cell therapy in solid tumors is significantly hampered by poor immune cell infiltration. The vascular endothelium can be considered a dynamic cellular organ and a barrier to CAR T-cell entry, which controls the passage of nutrients, maintains blood flow, and regulates leukocyte trafficking by controlling the chemokine and cytokine composition of the TME (58). Thus, chemokines play a vital role in tumor growth, remodeling, and T-cell trafficking to tumors in TME. For example, chemokines such as CXCL1 and CXCL12 are abundantly expressed and secreted by tumor cells and stromal cells. Also expressed are receptors such as CCR1-4, CCR9, and CX3CR1, which contribute to the entry of cancer cells into the vasculature and help them migrate and escape from effector T cells (59).

Moreover, previous studies have shown that neuroblastoma can secrete high levels of CCL2. The result in designing CAR-T cells to co-express CCR2b (the major isoform of the CCL2 chemokine receptor) showed that T cells homed to CCL2-expressing neuroblastoma and malignant pleural mesothelioma xenografts, respectively (60). Similarly, investigators firstly correlated CAR-T cells co-expressing CCR2b/CCR4 exhibited tumor total clearance *in vivo* to their higher tumor tissue homing activity (61). Indeed, the production of extracellular matrix (ECM) from tumor cells and cancer-associated fibroblasts (CAFs) would restrict the entry of T cells into the tumor, especially as high collagen density tumors display infiltrating T cells at lower levels. In this regard, CAR-T cells designed to express matrix-degrading enzymes, target CAFs, or disrupt tumor vasculature have demonstrated promising tumor infiltration of CAR-T cells (62, 63).

#### 3.2.2 T cell inhibitory signals

It is widely known that there are multiple inhibitory signals in the TME besides abundant immunosuppressive cells. PD-1, a typical T cell checkpoint, is expressed on activated T cells and, when bound by PD-1 ligand (PD-L1), inhibits the cytotoxicity of CAR-T cells and induces non-responsiveness. Thus, CAR-T cells must inhibit the PD-1/PD-L1 axis (64). Construction of CAR-T cells blocking the PD-1/PD-L1 pathway by short-hair RNA gene silencing technology (shRNA) for the treatment of lymphoma (CD19 antigen-expressing) and prostate (PSCA antigen-expressing) mice with subcutaneous xenografts showed remarkably longer survival time and reduced tumor volumes (65). Similarly, preclinical and clinical studies have shown that CRISPR/Cas9 gene editing systems are knocking down the PD-1 gene in CAR-T cells or combining immune checkpoint blockade with CAR-T cells can not only improve the ability of CAR-T cells to expand and reduce exhaustion but also increase the proportion of CD8^+^/CD4^+^ T cells in the TME (66, 67). In general, the efficacy of CAR-T cells can be improved by blocking antibodies, chimeric PD-1 switching molecules, or dominant-negative receptors. However, PD-1-deficient CAR-T cells are susceptible to CD8^+^ T cell exhaustion and lack long-term persistence (68). Therefore, continued research and clinical validation are needed in the future to fully understand the value of PD-1 knockdown or disruption for clinical applications.

#### 3.2.3 Hypoxic solid tumors

Hypoxia, the most prominent feature of TME, reduces the CD8^+^ T cells proliferation and anti-tumor function, preventing the development of anti-tumor immune responses. Recent studies have shown that a novel strategy of integrating the HIF structural domain to the intracellular domain of the CAR or introducing the HRE region on the promoter of the construct contributes to CAR hydroxylation and degradation in the presence of oxygen (69). Kosti et al. used tumor hypoxia as a physical cue for licensing CAR-T cell activation to establish HypoxiCAR T cells (a dynamic on/off oxygen sensing safety switch for CAR-T cells), which are safe and effective against solid tumors (70). Another attractive approach includes targeting antigens upregulated in hypoxic conditions (48) or restricting CAR expression to better-oxygenated environments (69). Contradictorily, others found that hypoxia enhanced the lytic activity and function of CD8^+^ T cells through an increase in granzyme B (71). In conclusion, the results of these early preclinical studies are essential for the future development of novel CAR-T cell therapies for the treatment of solid tumors.

#### 3.2.4 Safety of CAR-T cell

CAR-T cell therapy has achieved remarkable success in hematologic malignancies. Still, there is a need to improve safety and efficacy, overcome non-tumor toxicity, and optimize CAR design to expand the application of CAR-T cells (72, 73). As previously mentioned, the targeting structural domains of CAR-T cells are mainly based on single-chain antibodies, and peptide linkers are required between the heavy and light chain variable regions (74). After CAR-T cell infusion, the host immune system can mediate immune responses against such linkers by forming neutralizing antibodies due to the immunogenicity of the linkers. Recent studies have shown that nanobody-based CAR-T cells have significant antitumor effects (75). Nanosomes belong to the variable region of heavy chain antibodies (HcAbs) and contain only the variable regions of heavy chain and CH2 and CH3. Compared to mAb, which requires six complementarity-determining regions (CDRs) to bind antigens, nanobodies require only three CDRs with similar affinity and specificity (76). And the risk of immunogenicity is lower, making it safer than mAb derived from mice. Nanobodies also tend to have an advantage over single-chain antibodies in the context of humanization due to the more straightforward humanization process of nanobodies (77). In addition, T-cell failure may be associated with CAR aggregation on the CAR-T surface triggering activation of effector cells and cytotoxic signaling cascades. Nanobody-based CAR-T cells tend not to have the limitations of CAR surface aggregation and target antigen non-dependent effector cell activation (78).

Furthermore, other structural domains of the CARs can significantly affect the proliferation of CAR-T cells, the distribution of cytokines, and the side effects involving the treatment, including CRS, neurological problems, and On-Target-Off-Tumor Toxicity (79, 80). How to prevent these complications effectively and reliably in the future will be one of the critical factors of CAR-T cell therapy success.

## 4 Combination of radiotherapy with CAR-T cell therapy

Radioresistant CSCs after standard radiotherapy are probably among the significant causes of the recurrence of metastatic disease. It has been reported that RT combined with CAR-T cell therapy may be an effective strategy to prevent tumor recurrence after acquired radioresistance. Whether radioresistant tumor cells remain sensitive to CAR-T cells is an important question.

### 4.1 Preclinical and clinical data on RT plus CAR-T cell therapy

Developed as a promising strategy for overcoming the TME barriers, CAR-T cell therapy regimens likely hold applied value in irradiated TME. many clinical trials are being carried out (**Table 2**). In addition, Zhang et al. showed previously that fractionated irradiation (FIR) could upregulate immune checkpoint B7-H3 expression on bulk cells and radioresistant prostate cancer stem cells (PCSCs) (85). When the FIR and B7-H3 CAR-T cells were combined, they could target FIR-resistant PCSCs *in vivo*, eliminate immune checkpoint function, and mediate tumor cell lysis. Similarly, the switchable universal chimeric antigen receptor (UniCAR) system has been reported to mediate the secretion of relative proinflammatory cytokines and enhance T cell proliferation when targeting high-level radioresistant head and neck squamous cell carcinoma (HNSCC) *in vitro* (86, 87). Also, it inhibited the radiation-resistant cancer cells of immunodeficient mice *in vivo*. In GBM or other treatment-resistant primary cancers, CAR-T cells targeting CD133-positive tumor-initiating cells, a marker of radioresistance in multiple aggressive cancers, demonstrated superior efficacy (88). It is noteworthy that in radiation-resistant patients, the combination therapy should have immunostimulatory effects in addition to direct killing activity. Previous combination therapy with RT and exosomes derived from γδ-T cells (γδ-T-Exos) was shown to kill radiation-resistant nasopharyngeal carcinoma stem cell-like cells (NPC-CSCs) and maintain their cytotoxicity in immunosuppressive microenvironment (89).

**Table 2:** Clinical studies of RT in combination with CAR-T cells (ClinicalTrials.gov).

Disease	Study title	Treatment	Clinical trials.gov identifier & phase
Diffuse large B-cell lymphoma	CAR-T for R/R B-NHL	Radiation:20 × 2.0 Gy Drug: CD19/CD20/CD22 CAR- T	NCT03196830 Phase 1 (81)
Malignant Gliomas	Combination of Immunization and Radiotherapy for Malignant Gliomas	Radiation:3 × 2.0 Gy Drug: CAR-T or intracranial immunoadjuvant	NCT03392545 Phase 1 (82)
Multiple Myeloma	BCMA Targeted CAR T Cells With or Without Lenalidomide for the Treatment of Multiple Myeloma	Radiation:5 × 4.0 Gy Drug: EGFRt/BCMA-41BBz CAR T cell	NCT03070327 Phase 1 (83)
Multiple Myeloma	CART-BCMA Cells for Multiple Myeloma	Radiation:8 × 3.0 Gy Drug: BCMA CAR-T cell	NCT02546167 Phase 1 (84)
Multiple Myeloma	Phase II Study of Salvage Radiation Treatment After B-cell Maturation Antigen Chimeric Antigen Receptor T-cell Therapy for Relapsed Refractory Multiple Myeloma	Radiation:5 × 2.0 Gy Drug: BCMA CAR-T cell	NCT05336383 Phase 2 (Recruiting)

Additionally, low-dose radiation-exposed tumors present cells susceptible to TRAIL, a death-inducing ligand-mediated by RNA sequencing analysis (90). Through the study of pancreatic tumor cells *in vitro*, DeSelm et al. illustrated that TRAIL could be produced by CAR-T cells after binding to antigen-positive tumor cells, and the clearance of antigen-negative tumor cells has been previously exposed to a low dose of local or systemic radiation. Whole-body irradiation of complete lymphatic depletion is suggested to boost survival and proliferation *via* upregulation of IL-7 and IL-15 of CAR-T cells without competing with endogenous lymphocytes in the mouse model (91). In addition to the blood-brain barrier, the immunosuppression microenvironment is the main reason for the radioresistance of glioblastoma (GBM). In other mouse glioma models, CAR-T cells around the tumor site accumulated significantly after local subtherapeutic irradiation (12).

Overall, these studies successfully demonstrated that radioresistant cancer cells could be eradicated by combining CAR-T cells in a highly efficient and antigen-specific manner.

### 4.2 Challenges of CAR-T cells on radioresistant tumor cells

The focus of the successful implementation of radiotherapy combined with the CAR-T cell therapeutic approach is to analyze the effect of CAR-T cells on radioresistant tumor cells. On the one hand, radioresistance and tumor recurrence are mainly associated with CSCs and are considered targets for novel anticancer therapeutic agents (92). Identifying CSC-specific antigens for CAR-T cell targeting is a big hurdle; (i) target antigens should be expressed only on the surface of CSCs, not in their cytoplasm; (ii) target antigens should be expressed only by cancer cells, and particular CSC antigens should be selected to prevent targeted, non-tumor toxicity; (iii) selection of CSCs antigens expressed on common but tumor-specific antigens expressed on various tumor types, as some CSCs populations are characterized by a lack of cell surface expression of antigens (93). For example, leukemic cancer stem cells are characterized by CD34^+^CD38^-^ (94).

On the other hand, RT may cause an increase in Treg infiltration while increasing the enrichment of T cells into the tumor, and Treg infiltration may grow dose-dependent. Treg cells are more resistant to radiation than other T cell subsets, which may also be a potential obstacle to a strategy of combining RT with CAR-T cells (95). Furthermore, adenosine can be transformed by tumor cells releasing ATP and the ectoenzymes CD39 and CD73 after RT, resulting in increased adenosine expression that can be a barrier to the antitumor response of effector T cells with their surface A2a adenosine receptors (A2aRs). CRISPR-Cas9-based gene editing or engineered CAR-T cells to carry antagonist nanoparticles to deplete A2aRs in CAR-T cells (96). These results suggested that blocking adenosine signaling for adenosine-rich tumors mediated by RT can provoke a more effective T cells response in combination therapy.

### 4.3 Challenges of radiation dose and fractionation

The average tissue volume in routine RT is usually considerably more significant than the tumor volume itself due to the absence of dose delivery techniques that restrict regular tissue exposure in clinical practice. Therefore, applying fractionated irradiation regimens is the only alternative for providing high tumor doses (97). After specific doses and fractionated irradiation, CD8^+^ T cells and NK cells with antitumor effects were eliminated, whereas Tregs and MDSCs were left. A low dose of 2 Gy of radiation was reported to stimulate nitric oxide synthase through tumor-associated macrophages and generate an immunogenic environment. By contrast, high doses of radiation more significant than 5 to 10 Gy have been shown to promote severe vascular damage, limit the infiltration of CD8^+^ T cells into the tumor, and increase the area of hypoxia, which results in radioresistance (9). A 6 Gy irradiation dose can upregulate endothelial nitric oxide synthase (eNOS) expression and activity, which generates tumor angiogenesis and results in radioresistant suppressor cells recruitment, such as TAMs with the M2 phenotype, MDSC, and Tregs (98). In addition, numerous studies have proved that hypofractionated radiotherapy can medicate immune-activated TME and improve the therapeutic effect. At the same time, 7.5 Gy/fraction is recommended as the optimal fractionation regimen to induce an anti-tumor response (99). However, recent studies have shown that hypofractionated radiotherapy promotes immunosuppressive TME and plays an essential role in radioresistance and tumor recurrence (100). Indeed, there is a delicate balance between TME suppression and activation triggered by hypofractionated irradiation. The immune response may vary at each stage of the radiotherapy process depending on the dose, fractionation, tumor type, and site irradiation.

## 5 Conclusions and future perspectives

The successful CAR-T cell therapy for hematological malignancies signals a new era of immunotherapy in treating malignant diseases. Still, this review has pointed out emphatically that attaining effective clinical outcomes for radioresistant patients will require detailed consideration of this combination therapy. For example, according to the description of Sim et al., in the case of patients receiving CAR-T cell therapy, adequate local disease control before CAR-T cell infusion could cause the lowest acute toxicity (101). In addition to photons, proton irradiation has emerged as an approach to increase CSC sensitivity from various tumor cell lines (102). But, the optimal or proper dose, the time of fractionation and radiation exposure, and the time of CAR-T cell infusion are still unknown. Notably, a clinical trial (NCT02546167) showed mild acute RT-related toxicity in two patients treated with combination therapy (103). Therefore, more extensive clinical studies must clarify how RT affects CAR-T’s toxicity and efficacy through immunological mechanisms. The current widely recognized mechanism is that as CARs cause activation of MHC-independent T cells, CAR-T cells can secrete cytokines in response to RT may lead to an abscopal-like in endogenous T cells; RT-mediated apoptosis of tumor cells results in antigens release eventually presented through APCs, which may stimulate the clonal expansion of CAR-T cells and endogenous T cells.

**Figure 4:**
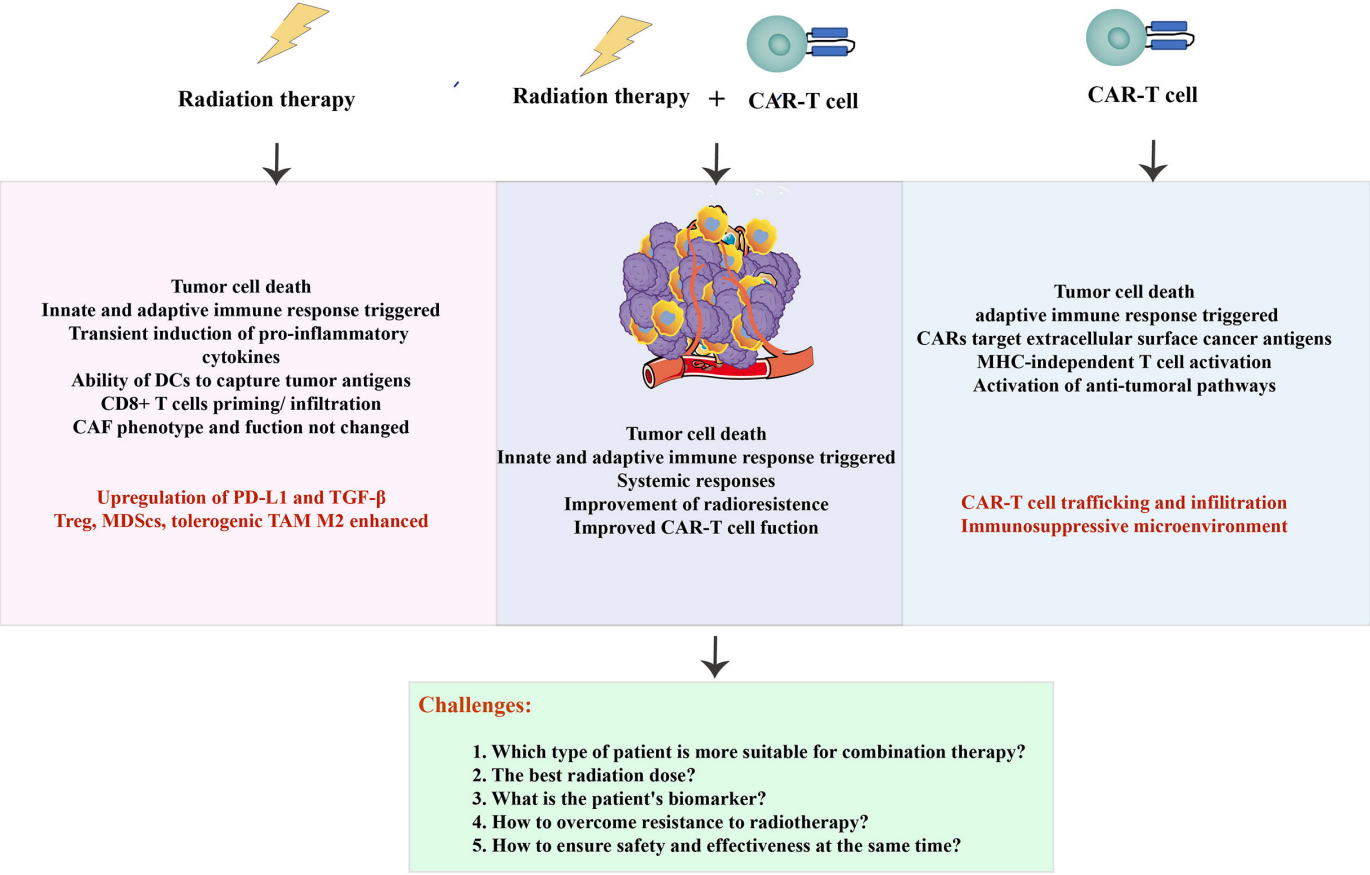
Summarized schematic. The advantages and challenges of radiotherapy and CAR-T cells (in red), as well as the benefits and problems to be solved in combination therapy.

Additionally, CAR-T cells are subject to a complex immunosuppressive microenvironment in radioresistant patients, which has hampered progress. Most tumor treatments failed and relapsed due to individual immunity and comorbidity conditions. Therefore, an integration of next-generation sequencing technologies provides newer opportunities to understand the dynamic antigen landscape and other immune-related factors of tumor cells in the TME. In terms of RT combination with CAR-T cell therapy, at least for now, those are lofty goals that are far, far away (**Figure 4**).

## Author contributions

TH researched data and wrote the article. HL and BT contributed to scientific discussion and critical review of the manuscript. Contributions to the article were made by all three authors, and the submitted version was approved.

## Funding

Health Research Project of Huai’an City in 2020, General Program, Jiangsu, China. Grant number 33.

## Acknowledgments

We would like to thank Jinhu County People’s Hospital and Jiangsu University for their support.

## Conflict of interest

The authors declare that the research was conducted in the absence of any commercial or financial relationships that could be construed as a potential conflict of interest.

## Publisher’s note

All claims expressed in this article are solely those of the authors and do not necessarily represent those of their affiliated organizations, or those of the publisher, the editors and the reviewers. Any product that may be evaluated in this article, or claim that may be made by its manufacturer, is not guaranteed or endorsed by the publisher.
